# Developing a competency framework for operating room nurse specialist training instructors: a Delphi study

**DOI:** 10.3389/fsurg.2026.1877571

**Published:** 2026-09-10

**Authors:** Jiye Sang, GuoJun Zhao, Mingming Zhao, Jizhong Li, Yanhong Zhang, Xiaoting Geng

**Affiliations:** 1 Affiliated Hospital of Chengde Medical University, Operating Room, Chengde City, China; 2 Affiliated Hospital of Chengde Medical University, Nursing Department, Chengde City, China; 3 Affiliated Hospital of Chengde Medical University, Ophthalmology, Chengde City, China

**Keywords:** analytic hierarchy process, clinical instructor, competency, Delphi technique, nurse specialist, operating room

## Abstract

**Objective:**

To develop a competency evaluation framework for OR nurse specialist training instructors.

**Methods:**

Guided by the Iceberg Model and the Onion Model of competency, a preliminary indicator framework was developed through a literature review, behavioral event interviews, and expert group discussions. A two-round Delphi survey involving 31 operating room nursing experts was conducted to refine the indicators. The Analytic Hierarchy Process (AHP) was used to determine the weights of the final indicators. Expert authority, participation rate, and the degree of consensus were assessed.

**Results:**

A competency evaluation framework consisting of five first-level indicators and 36second-level indicators was established. The expert authority coefficients in the two rounds were 0.829 and 0.830, respectively. Kendall’s W coordination coefficients were *0.157 and 0.112* (both *P* < 0.05). The weights of the first-level indicators, ranked from highest to lowest, were Professional Knowlege (41.10%), Research Capability (20.68%), Teaching Ability (18.33%), Professionalism (13.79%), and Personality Traits (6.10%).

**Conclusion:**

The resulting framework demonstrates sound validity and reliability. It provides a structured basis for the selection, training, and assessment of OR nurse specialist instructors, which may support efforts to improve specialist nursing education quality.

## 1 Introduction

The global healthcare landscape is undergoing a profound transformation, driven by rapid technological advances, an increasing emphasis on value-based care, and rising patient expectations regarding safety and specialized expertise (1–3). Against this backdrop, specialized nursing practice has assumed an increasingly important role in delivering high-quality, patient-centered care (4, 5).

Operating room (OR) nursing exemplifies this specialization, functioning within a uniquely high-risk environment characterized by inherent clinical risks, stringent aseptic requirements, rapid technological innovation, and the imperative to achieve optimal surgical outcomes. The increasing complexity of modern surgical procedures requires a nursing workforce whose competencies extend beyond basic clinical skills to encompass advanced professional knowledge, refined technical expertise, sound critical thinking, and effective interdisciplinary collaboration (6, 7). Accordingly, systematic education and training of OR nurse specialists are essential for ensuring patient safety, improving procedural efficiency, and enhancing the overall quality of perioperative care.

The quality of this specialized nursing workforce depends largely on the competence of those responsible for its education and training. Instructors responsible for preparing OR nurse specialists play a dual role: they must possess both advanced clinical expertise acquired through extensive operating room experience and the pedagogical competence to translate complex clinical practice into effective learning experiences (8–10). Their competencies directly influence trainees’ clinical judgment, technical proficiency, and professional values, thereby exerting a cascading influence on the quality of perioperative care across healthcare institutions. Despite broad recognition of the critical role of instructors, systematic evaluation of their competencies remains insufficiently explored.

Existing research on competencies in the OR nursing field has primarily focused on two areas: the development of competency models and assessment instruments for practicing OR nurses, and the design of curricula and training programs for OR nurse specialists. Although these studies have made valuable contributions, they have largely overlooked the specific competency requirements of instructors responsible for delivering specialist training. Moreover, although several general competency frameworks for nurse educators have been established, they often lack the contextual specificity required for the operating room environment. The unique demands of surgical practice—including sterile technique, surgical instrumentation, patient positioning, hemodynamic monitoring, and crisis management—require specialized knowledge, skills, and professional attributes that generic educator competency frameworks may not adequately capture (11, 12). Therefore, an important gap remains: the absence of a standardized, context-specific, and empirically validated instrument for comprehensively evaluating the competencies of OR nurse specialist training instructors.

Accordingly, this study aimed to develop and validate a comprehensive competency evaluation index system specifically designed for instructors responsible for OR nurse specialist training. Drawing upon established competency theory and employing rigorous consensus-building methods, this study developed a structured and weighted evaluation framework that reflects the multifaceted responsibilities of these educators. The resulting framework is expected to provide an evidence-based tool for healthcare institutions and nursing education organizations to support instructor selection, guide professional development, inform performance evaluation, and ultimately improve the quality and standardization of OR nurse specialist training.

## 2 Methods

### 2.1 Study design

This study employed a sequential exploratory mixed-methods design. The study comprised two sequential phases: (1) a qualitative phase involving item generation through a literature review and behavioral event interviews, and (2) a quantitative phase involving expert consensus building and indicator weighting using the Delphi method and the Analytic Hierarchy Process (AHP).

### 2.2 Development of the preliminary framework

The preliminary competency framework was developed through a sequential process involving a literature review, behavioral event interviews, and expert group discussions. First, a comprehensive literature review was conducted to identify competency domains and indicators reported in the literature on nurse educators and perioperative nursing. Subsequently, behavioral event interviews were conducted to explore the competencies associated with effective OR nurse specialist training instructors. The preliminary competency framework was then refined through expert group discussions before being submitted for Delphi consultation.

#### 2.2.1 Literature review

A systematic literature search was conducted to identify existing competency frameworks and competency indicators relevant to operating room (OR) nurse specialist training instructors. The search was conducted in three electronic databases: PubMed, CINAHL (via EBSCO), and the China National Knowledge Infrastructure (CNKI). All publications from database inception to December 2024 were searched.

The PubMed search strategy was as follows:

(“Nurse Educators"[MeSH] OR “nurse educator*” OR “clinical instructor*” OR “preceptor*” OR “trainer*”) AND (“competenc*"[Title/Abstract] OR “capability*"[Title/Abstract] OR “skill*"[Title/Abstract] OR “qualification*"[Title/Abstract]) AND (“operating room"[Title/Abstract] OR “perioperative nursing"[Title/Abstract] OR “surgical nursing"[Title/Abstract] OR “intraoperative care"[Title/Abstract]).

The CINAHL search strategy was as follows:

(MH “Nurse Educators+”) OR “nurse educator*” OR “clinical instructor*” OR “preceptor*” AND (“competenc*” OR “capability*” OR “skill*” OR “qualification*”) AND (“operating room” OR “perioperative nurs*” OR “surgical nurs*”).

The inclusion criteria were as follows: (a) peer-reviewed journal articles or official reports; (b) publications in either English or Chinese; (c) studies focusing on competency frameworks, competency indicators, or role requirements for nurse educators or preceptors, particularly in perioperative settings; and (d) studies reporting original research or established competency frameworks, including Delphi studies, literature reviews, and qualitative studies.

The exclusion criteria were as follows: (a) conference abstracts, editorials, commentaries, and other non-peer-reviewed publications; (b) studies focusing exclusively on general clinical competence without educator-specific competency dimensions; and (c) duplicate publications or articles with incomplete full texts. Two reviewers independently screened titles and abstracts, followed by full-text review of potentially eligible studies. Any disagreements were resolved through discussion, with consultation from a third reviewer when necessary consultation with a third reviewer.

#### 2.2.2 Behavioral event interviews (BEI)

Semi-structured Behavioral Event Interviews (BEIs) were conducted with eight experienced operating room (OR) nurse educators and nursing managers to identify critical job behaviors and the competencies required for OR nurse specialist training instructors. Participants were recruited through purposive sampling. Eligible participants were required to have at least 10 years of OR nursing experience and current or recent experience in OR nursing education or management. The sample size was determined according to the principle of data saturation, which is widely applied in qualitative research.

An interview guide was developed based on a review of the relevant literature and informed by the Iceberg Model and the Onion Model of competency. Each interview lasted approximately 45–60 min and was conducted face-to-face in a private interview room. With participants’ informed consent, all interviews were audio-recorded and transcribed verbatim. Field notes were recorded during and immediately after each interview to document contextual information and researchers’ observations.

Interview data were analyzed using Braun and Clarke’s six-phase thematic analysis approach. Two researchers independently coded the transcripts using an initial coding framework informed by the Iceberg Model and the Onion Model of competency. Coding discrepancies were resolved through discussion until consensus was achieved. Themes and subthemes were iteratively refined, and representative quotations were selected to illustrate key competency domains. The analysis identified a set of core competencies encompassing both surface-level competencies (knowledge and skills) and deeper-level competencies, including professional attitudes, motivation, self-concept, and personal characteristics.

#### 2.2.3 Expert group discussion

A preliminary competency framework was developed based on the findings of the literature review and Behavioral Event Interviews (BEIs). The preliminary framework was subsequently reviewed and refined through expert group discussions involving a seven-member research team consisting of two nursing administrators, two nursing education experts, two operating room nursing managers, and one graduate student with expertise in research methodology. The research team evaluated the clarity, relevance, and comprehensiveness of each competency indicator and revised the framework through iterative discussions until consensus was achieved. The resulting preliminary framework served as the basis for the subsequent Delphi consultation.

### 2.3 Expert panel

Thirty-one experts were recruited through purposive sampling from the expert database of the Operating Room Nursing Committee of the Chinese Nursing Association. The inclusion criteria were as follows: (1) holding an associate senior professional title or a higher academic rank; (2) having at least 8 years of experience in operating room nursing or nursing education; (3) currently serving as an OR nurse specialist instructor or nursing educator; and (4) providing informed consent and agreeing to participate voluntarily. The characteristics of the expert panel are presented in Table 1.

**Table 1:** Basic information of the expert panel (*n* = 31).

Characteristic	Category	*n*	%
Gender	Male	4	12.9
	Female	27	87.1
Age (years)	30−39	9	29.03
	40−49	11	35.48
	≥50	11	35.48
Education level	Bachelor’s degree	19	61.29
	Master’s degree	9	29.03
	Doctorate	3	9.68
Professional field	Operating room nursing	18	58.06
	Surgical nursing	3	9.68
	Nursing management	4	12.90
	Clinical nursing education	6	19.35
Working experience (years)	8−12	6	19.35
	13−19	6	19.35
	≥20	19	61.29

### 2.4 Delphi process

A two-round Delphi survey was conducted to establish expert consensus on the competency evaluation framework. The Delphi questionnaires were distributed electronically via email and the Wenjuanxing online survey platform. Experts completed the questionnaires independently and returned them within 2 weeks. Reminder emails were sent 1 week after the initial distribution to improve the response rate.

The first-round questionnaire was developed from the preliminary competency framework and employed a 5-point Likert scale (1 = very unimportant, 5 = very important) to assess the importance of each indicator. Experts were encouraged to provide suggestions regarding the modification, addition, or deletion of indicators.

The second-round questionnaire incorporated controlled feedback from the first round, including the mean score, standard deviation (SD), and coefficient of variation (CV) for each indicator, together with the revised indicator list.

Consensus was considered to be achieved when an indicator met all of the following criteria: a mean importance score ≥ 3.5, a coefficient of variation (CV) < 0.25, and a full-score ratio > 20%. Indicators that did not satisfy these criteria were reviewed by the research team for revision or deletion.

### 2.5 Data analysis

Data were analyzed using SPSS version 22.0 (IBM Corp., Armonk, NY, USA) and Yaahp AHP software. Descriptive statistics were used to summarize the characteristics of the expert panel and the ratings assigned to each competency indicator. Expert participation was evaluated according to the effective questionnaire response rate.

Expert authority was assessed using the authority coefficient (Cr), which was calculated based on the judgment basis coefficient (Ca) and the familiarity coefficient (Cs). The judgment basis coefficient (Ca) was derived from four sources: theoretical analysis, practical experience, literature references, and intuitive judgment. The familiarity coefficient (Cs) was determined according to experts’ self-rated familiarity with the research topic. The authority coefficient was calculated as:

Cr = (Ca + Cs)/2.A Cr value of ≥ 0.70 was considered indicative of acceptable expert authority.

Indicators that achieved consensus were subsequently incorporated into the Analytic Hierarchy Process (AHP) model. A hierarchical structure was established, and pairwise comparison matrices were constructed using the mean importance scores obtained in the second Delphi round. Because of the large number of indicators, Saaty scale values were assigned objectively according to the differences in mean importance scores between paired indicators. Specifically, if d represented the difference in mean importance scores, the corresponding Saaty scale values were assigned as follows: 1 (d = 0), 2 (0 < d ≤ 0.25), 3 (0.25 < d ≤ 0.50), 4 (0.50 < d ≤ 0.75), 5 (0.75 < d ≤ 1.00), 6 (1.00 < d ≤ 1.25), 7 (1.25 < d ≤ 1.50), 8 (1.50 < d ≤ 1.75), and 9 (d > 1.75). Pairwise comparison matrices were then constructed accordingly. The consistency ratio (CR) was calculated for each judgment matrix, with CR < 0.10 indicating acceptable consistency. Local and global priority weights were subsequently calculated.

### 2.6 Ethical approval

This study received ethical approval from the Ethics Committee of the Affiliated Hospital of Chengde Medical University (Approval No. LL2021085; June 28, 2021). All participants were informed of the study objectives and procedures before enrollment. Participation was entirely voluntary, and written informed consent was obtained from all experts participating in the Behavioral Event Interviews and Delphi survey. Participant anonymity and confidentiality were strictly protected throughout the study, and no personally identifiable information was collected or reported.

## 3 Results

### 3.1 Response rates and expert participation

In the first Delphi round, Thirty-one questionnaires were distributed, and 27 valid questionnaires were returned, yielding an effective response rate of 87.1%. Among the respondents, 26 experts (83.9%) provided comments and suggestions for revising the competency indicators. In the second Delphi round, questionnaires were distributed to the 27 experts who had completed the first round. All questionnaires were returned and deemed valid, yielding a 100% response rate. Ten experts (37.0%) proposed additional revisions to the competency framework. The response rates and level of expert participation in the two Delphi rounds are presented in Table 2.

**Table 2:** Enthusiasm level of the experts.

Round	Effective response rate (%)	Proportion offering suggestions (%)
First	87 (27/31)	83.87 (26/31)
Second	100 (27/27)	37.03 (10/27)

### 3.2 Expert authority

The expert authority coefficient (Cr), calculated from the judgment basis coefficient (Ca) and familiarity coefficient (Cs), was used to assess the authority of the expert panel. The Cr values for the first and second Delphi rounds were 0.829 and 0.830, respectively. According to established Delphi methodology, a Cr value of ≥ 0.70 indicates acceptable expert authority and the reliability of expert judgments. Therefore, the expert panel in this study demonstrated a high level of authority, supporting the credibility of the Delphi consultation results (13–15).

### 3.3 Kendall coordination

Kendall’s coefficient of concordance (W) was used to assess the degree of agreement among the experts. The Kendall’s W values for the first and second Delphi rounds were 0.157 and 0.112, respectively. Both coefficients were statistically significant (*P* < 0.05), indicating a statistically significant level of agreement among the experts. The detailed results are presented in Table 3.

**Table 3:** Kendall’s coefficient of concordance for expert opinions.

Round	*χ*2	*P*	W
First	138.729	0.000	0.157
Second	133.849	0.000	0.112

### 3.4 Development of the competency indicator system and indicator weights

The first-round expert consultation questionnaire included 4 first-level indicators and 23 s-level indicators. After the expert consultation, the indicator system was revised as follows:

1. “Personality Traits” was added as a first-level indicator.
2. Under the first-level dimension of “Professional Skills”, “Anatomy Knowledge”, “Pedagogical Knowledge”, and “PPT Creation Skills” were added, and added “Critical Thinking” and “Learning Ability” were moved to the “Personality Traits” dimension.
3. Under the “Research Capability” dimension, “English Literature Reading Ability”, “Grant Proposal Writing Ability”, and “Statistical Software Proficiency” were added, and “Literature Review Ability” was changed to “Literature Retrieval Ability”.
4. Under the “Teaching Ability” dimension, “Multimedia Teaching Ability” was added, and “Health Education Ability” was moved to the “Professional Skills” dimension.
5. Under “Professionalism”, the item “Care for Students” was added.
6. Under the “Personality Traits” dimension, “Self-Confidence”, “Emotion Control”, “Teamwork”, “Approachability”, and “Communication” were included.

Based on statistical analysis and group discussion, all suggestions provided by the experts were incorporated into the final system. The second-round questionnaire was compiled, including 5 first-level and 36 s-level indicators.

In the second-round questionnaire, experts collectively proposed 4 suggestions for revision:

1. Change “Anatomy Knowledge” to “Regional Anatomy Knowledge”.
2. Merge “Communication Ability” and “Communication” into a single item called “Communication Ability”.
3. Under the first-level indicator “Teaching Ability”, delete “Educational Knowledge” and move it under “Professional Competence”.
4. “Communication Ability” moved from the Personality Traits dimension to Professionalism.

The final indicator system is shown in Table 4.

**Table 4:** Evaluation indicators.

Dimension	Competency elements	Mean ± SD	CV%	Full score Ratio (%)	Combined weight (%)	Weight (%)
A Professional		4.90 ± 0.30	6.13%	90.32%	41.10%	
knowledge	A1 Humanistic Nursing Knowledge	4.71 ± 0.53	11.23%	74.19%	9.34%	3.84%
	A2 Professional Practice in OR	4.65 ± 0.66	14.22%	74.19%	7.23%	2.97%
	A3 Specialized Nursing Experience in OR	4.74 ± 0.58	12.14%	80.65%	11.22%	4.61%
	A4 Critical Thinking Ability	4.77 ± 0.50	10.42%	80.65%	11.79%	4.85%
	A5 Psychologica Knowledge	4.84 ± 0.45	9.39%	87.10%	15.23%	6.26%
	A6 Knowledge of Medica Devices	4.81 ± 0.48	9.93%	83.87%	13.15%	5.40%
	A7 Knowledge of Medica Equipment	4.68 ± 0.48	10.16%	67.74%	8.30%	3.41%
	A8 Professional OR Nursing Knowledge	4.45 ± 0.57	12.76%	48.39%	4.78%	1.96%
	A9 Frontier Knowledge in OR Nursing Writing Ability	4.42 ± 0.67	15.21%	51.61%	4.02%	1.65%
	A10 Surgical Knowledge	4.58 ± 0.56	12.32%	61.29%	5.84%	2.40%
	A11 Anatomy Knowledge	4.19 ± 0.83	19.87%	41.94%	2.74%	1.13%
	A12 Pedagogical knowledge	4.35 ± 0.71	16.29%	48.39%	2.84%	1.17%
	A13 Knowledge of using PPT and related software	4.39 ± 0.72	16.31%	51.61%	3.53%	1.45%
B Research capability		4.68 ± 0.54	11.56%	70.97%	20.68%	
	B1 Problem Identiﬁcation Ability	4.48 ± 0.93	20.66%	67.74%	7.41%	1.53%
	B2 Literature Retrieva Ability	4.52 ± 1.03	22.78%	77.42%	8.86%	1.83%
	B3 Research Design Ability	4.68 ± 0.54	11.56%	70.97%	15.98%	3.30%
	B4 Statistical Analysis Ability	4.55 ± 0.72	15.89%	67.74%	10.50%	2.17%
	B5 Scientiﬁc Writing Ability	4.61 ± 0.62	13.34%	67.74%	14.86%	3.07%
	B6 English Literature Reading Ability	4.71 ± 0.53	11.23%	74.19%	18.69%	3.86%
	B7 Statistical Software Proﬁciency	4.45 ± 0.62	14.02%	51.61%	8.95%	1.85%
	B8 Grant Proposal	4.58 ± 0.50	10.95%	58.06%	14.76%	3.05%
C Teaching ability		4.61 ± 0.62	13.34%	67.74%	18.33%	
	C1 Theoretical Lecturing Ability	4.87 ± 0.43	8.78%	90.32%	42.93%	7.87%
	C2 Clinical Teaching & Supervision Ability	4.55 ± 0.68	14.85%	64.52%	21.56%	3.95%
	C3 Teaching Planning & Organizing Ability	4.68 ± 0.65	13.95%	77.42%	26.61%	4.88%
	C4 Multimedia Teaching Ability	4.39 ± 0.62	14.02%	45.16%	8.91%	1.63%
D Professionalism		4.52 ± 0.68	14.99%	61.29%	13.79%	
	D1 Devotion to Position	4.65 ± 0.55	11.85%	67.74%	10.55%	1.45%
	D2 Dedication Spirit	4.90 ± 0.30	6.13%	90.32%	30.92%	4.26%
	D3 Positivity & Optimism	4.68 ± 0.48	10.16%	67.74%	14.12%	1.95%
	D4 Prudent & Honest Self-Discipline	4.77 ± 0.56	11.74%	83.87%	19.38%	2.67%
	D5 *Communication Ability*	4.77 ± 0.50	10.42%	80.65%	19.38%	2.67%
	D6 Care for Students	4.35 ± 0.49	11.17%	35.48%	5.66%	0.78%
E Personality traits		3.42 ± 0.68	13.99%	38.71%	6.10%	
	E1 Self-Conﬁdence	4.55 ± 0.62	13.72%	61.29%	35.36%	2.16%
	E2 Emotion Control	4.48 ± 0.63	13.95%	54.84%	23.04%	1.40%
	E3 Teamwork	4.48 ± 0.68	15.09%	58.06%	23.04%	1.40%
	E4 Approachability	4.23 ± 0.72	16.97%	38.71%	10.60%	0.65%

## 4 Discussion

### 4.1 Reliability and scientific validity of the competency evaluation indicator system

This study developed a competency evaluation indicator system for OR nurse specialist training instructors through a systematic literature search, Behavioral Event Interviews (BEIs), and a two-round Delphi survey. These methods are widely recognized as rigorous approaches for developing consensus-based competency frameworks in healthcare research (16). Guided by the Onion Model and the Iceberg Model of competency, potential competency domains were initially identified through literature analysis and qualitative interviews. These findings were subsequently integrated with the job characteristics and competency requirements of OR nurse specialist training instructors to develop a preliminary competency framework.

The Delphi process enabled the systematic integration of expert opinions from multiple institutions and regions. The expert panel comprised individuals with extensive experience in operating room nursing, nursing education, and clinical management, thereby enhancing the credibility of the consultation results. Throughout the two consultation rounds, expert feedback was systematically reviewed and incorporated through iterative revisions, resulting in the progressive refinement of the competency indicator system.

The statistical findings further support the reliability and content validity of the developed indicator system. The expert familiarity coefficient, judgment coefficient, and authority coefficient were 0.91, 0.85, and 0.88, respectively, indicating a high level of expert authority. In addition, the effective response rates of 87% and 100% in the first and second Delphi rounds, respectively, indicate a high level of expert engagement throughout the consultation process.

The coefficient of variation for all indicators ranged from 0 to 0.17, indicating limited variability in experts’ ratings of individual indicators. Furthermore, Kendall’s coefficient of concordance was statistically significant in both Delphi rounds (W₁ = 0.157, *χ*² = 175.3, df = 36, *P* < 0.001; W₂ = 0.112, *χ*² = 125.4, df = 36, *P* < 0.001), demonstrating a statistically significant level of agreement among the experts. Although the Kendall’s W values indicate only slight overall agreement, this finding is not unexpected given the relatively large number of competency indicators and the heterogeneous backgrounds of the expert panel. Previous studies have shown that Kendall’s W tends to decrease as the number of evaluation items increases, even when consensus has been achieved according to predefined Delphi criteria (17). Moreover, the consistently low coefficients of variation indicate strong agreement regarding the importance of individual indicators. Collectively, these findings suggest that the developed competency evaluation indicator system demonstrates satisfactory reliability and content validity and provides a sound basis for its application in the evaluation and development of OR nurse specialist training instructors.

### 4.2 Characteristics of the competency evaluation indicators for operating room nurse specialist training instructors

The final competency evaluation indicator system comprised five first-level competency dimensions and 36 s-level indicators. Among the five dimensions, Professional Knowledge received the highest weighting (41.10%), followed by Research Capability (20.68%), Teaching Ability (18.33%), Professionalism (13.79%), and Personality Traits (6.10%). These findings suggest that professional knowledge is regarded as the most important competency for operating room (OR) nurse specialist training instructors.

Notably, Research Capability received a slightly higher weighting than Teaching Ability, a finding that merits particular attention. This result is consistent with previous studies reporting that research competence and evidence-based practice have become increasingly important attributes of advanced nursing educators, reflecting the growing emphasis on academic scholarship in specialist nursing education (18). Similarly, previous research has shown that the ability to conduct, interpret, and apply research distinguishes expert clinical instructors from competent practitioners (19). One possible explanation is that OR nurse specialist training instructors are expected not only to deliver high-quality clinical teaching but also to promote evidence-based practice, evaluate clinical innovations, and mentor trainees in scholarly inquiry. By comparison, teaching ability may be regarded as a foundational competency expected of all qualified instructors, resulting in a relatively lower weighting during the pairwise comparison process.

The high weighting assigned to Professional Knowledge is consistent with previous Delphi studies demonstrating that clinical expertise forms the foundation of specialist nursing education (20). This consistency with earlier research further supports the content validity of the developed competency framework. The indicator system was developed with consideration of both the educational characteristics of specialist nurse training and the clinical responsibilities of OR nurses. Consequently, it captures the key competencies required for OR nurse specialist training instructors while distinguishing between foundational competencies (Professional Knowledge, Research Capability, and Teaching Ability) and differentiating competencies (Professionalism and Personality Traits), which may help identify outstanding instructors beyond minimum professional requirements.

#### 4.2.1 Professional knowledge

Professional Knowledge was identified as the highest-priority competency dimension, highlighting the importance of clinical expertise in specialist nurse education. OR nurse specialist training instructors perform dual roles as clinicians and educators; therefore, their professional knowledge directly influences the quality of clinical teaching and specialist nurse training. A comprehensive understanding of perioperative nursing, surgical procedures, patient management, and specialized technical skills is essential for ensuring safe, high-quality perioperative care (21, 22).

Furthermore, OR nursing is inherently practice-oriented, requiring instructors to integrate theoretical knowledge with extensive clinical experience. Competence in surgical procedures, intraoperative nursing, and multidisciplinary collaboration enables instructors to translate complex clinical concepts into meaningful learning experiences. Such integration is essential for fostering trainees’ clinical reasoning, technical competence, and ability to apply evidence-based knowledge in perioperative practice (23).

#### 4.2.2 Teaching ability

Teaching Ability was identified as a core competency, emphasizing the educational responsibilities of OR nurse specialist training instructors. Effective teaching requires instructors to design learner-centered curricula, organize educational activities systematically, and employ appropriate instructional strategies that facilitate knowledge acquisition and professional skill development (18, 24).

In addition to subject expertise, instructors should be able to translate complex clinical concepts into understandable learning experiences by linking theoretical knowledge with authentic clinical practice. Educational approaches such as case-based learning, simulation-based education, and clinical demonstrations have been shown to enhance clinical reasoning, problem-solving ability, and critical thinking among nursing trainees (25–28).

The increasing adoption of multimedia-supported teaching, online learning, and blended educational models has further expanded the competency requirements of nurse educators. Previous studies suggest that multimedia technologies can improve learning engagement and facilitate understanding of complex clinical concepts (29, 30). Consequently, OR nurse specialist training instructors should possess competencies in instructional design, digital pedagogy, and the effective integration of educational technologies into specialist nurse training.

#### 4.2.3 Professionalism and personality traits

Professionalism and Personality Traits represent differentiating competency dimensions that reflect deeper personal attributes, including professional values, ethical responsibility, motivation, emotional stability, and self-concept. Competency theory suggests that these characteristics often distinguish outstanding performers from competent practitioners by influencing long-term professional performance and career development.

Within clinical education, instructors’ professional attitudes and personal characteristics substantially influence students’ learning experiences and professional socialization. Attributes such as integrity, dedication, teamwork, emotional regulation, and effective communication contribute to supportive learning environments and positive teacher–student relationships. Therefore, evaluating these dimensions alongside clinical expertise and teaching competence may provide a more comprehensive assessment of instructor performance.

By incorporating these differentiating competencies, the proposed framework extends beyond the evaluation of technical and educational competence to include the personal attributes that support excellence in clinical education. This comprehensive approach may facilitate the selection, development, and evaluation of high-quality OR nurse specialist training instructors and contribute to the continued advancement of perioperative nursing education.

## 5 Conclusion

This study developed and validated a competency evaluation indicator system for operating room nurse specialist training instructors using a systematic literature search, Behavioral Event Interviews, the Delphi method, and the Analytic Hierarchy Process. The final framework comprises five competency dimensions—Professional Knowledge, Research Capability, Teaching Ability, Professionalism, and Personality Traits—and 36 weighted secondary indicators.

The findings indicate that Professional Knowledge, Research Capability, and Teaching Ability constitute the core competencies required for OR nurse specialist training instructors, while Professionalism and Personality Traits provide additional differentiating competencies that distinguish high-performing educators. The resulting framework reflects the dual clinical and educational responsibilities of OR nurse specialist training instructors and offers a structured approach to competency evaluation.

This competency evaluation framework may provide nursing administrators and educational institutions with an evidence-based tool to support instructor selection, performance evaluation, professional development, and faculty training. Furthermore, its application may contribute to improving the quality and standardization of specialist nurse education and support the development of a highly competent perioperative nursing workforce.

## 6 Limitations and future directions

This study has several limitations that should be acknowledged. First, the generalizability of the proposed competency evaluation framework is limited by the composition of the expert panel. All experts were recruited from the expert database of the Operating Room Nursing Committee of the Chinese Nursing Association. Consequently, the framework primarily reflects the characteristics of the Chinese healthcare system and nursing education context and may require contextual adaptation before application in other countries or healthcare settings.

Second, although the Delphi method provides a rigorous and structured approach to achieving expert consensus, it remains dependent on expert judgment, which may introduce subjective bias despite the iterative consultation process. Furthermore, the competency framework was developed through expert consensus and has not yet undergone large-scale empirical validation in clinical or educational settings. In addition, the proposed competency evaluation framework has not yet been implemented in routine educational practice. Therefore, its feasibility, usability, inter-rater reliability, and responsiveness in real-world training settings remain to be established.

Future research should focus on three main directions. First, cross-cultural validation should be prioritized by involving international experts and evaluating the framework across diverse healthcare systems to improve its generalizability and identify potential contextual differences. Second, psychometric validation using structural equation modeling (SEM) and other quantitative approaches is needed to examine the construct validity, reliability, and predictive validity of the framework in relation to instructor performance and trainee outcomes. Furthermore, implementation studies are warranted to evaluate the feasibility, usability, inter-rater reliability, and responsiveness of the framework in routine educational practice. Finally, longitudinal studies should be conducted to monitor changes in instructor competency over time and evaluate the long-term effectiveness of the framework in supporting instructor development and enhancing the quality of specialist nurse education.

## Data Availability

The datasets presented in this article are not readily available because due to the nature of the Delphi method, the raw dataset contains identifiable expert demographic information and individual rating scores that could potentially reveal participants’ identities. Therefore, the data are not publicly available to protect participant confidentiality and comply with ethical approval requirements. De-identified summary data supporting the findings are available from the corresponding author upon reasonable request. Requests to access the datasets should be directed to JiyeSANG 1057067874@qq.com.
